# Increasing Diversity in Developmental Biology

**DOI:** 10.3389/fsoc.2021.762836

**Published:** 2022-02-02

**Authors:** Graciela A. Unguez, Karen L. Bennett, Carmen Domingo, Ida Chow

**Affiliations:** ^1^ Biology, New Mexico State University, Las Cruces, NM, United States; ^2^ School of Medicine, University of Missouri, Columbia, MO, United States; ^3^ San Francisco State University, San Francisco, CA, United States; ^4^ Society for Developmental Biology, Rockville, MA, United States

**Keywords:** diversity and inclusion, developmental biology, undergraduate research, choose development, summer research program

## Abstract

The demographic profile of the scientific and biomedical workforce in the United States does not reflect the population at large (https://ncses.nsf.gov/pubs/nsf21321/data-tables; www.census.gov), raising concerns that there will be too few trained researchers in the future, the scope of research interests will not be broad enough, gaps in equity and social justice will continue to increase, and the safeguards to the integrity of the scientific enterprise could be jeopardized. To diversify the pool of scientists, the Society for Developmental Biology (SDB) developed the *Choose Development!* Program—a two-summer immersion for undergraduate students belonging to underrepresented (*UR*) populations in STEM to join the research laboratory of an established SDB member. This research-intensive experience was augmented by a multi-tier mentoring plan for each student, society-wide recognition, professional development activities and networking at national meetings. The strengths of the *Choose Development!* Program were leveraged to expand inclusion and outreach at the Society’s leadership level, the Board of Directors (BOD), which then led to significant changes that impacted the SDB community. The cumulative outcomes of the *Choose Development!* Program provides evidence that community-based, long-term advocacy, and mentoring of young *UR* scientists is successful in retaining *UR* students in scientific career paths and making a scientific society more inclusive.

## Background

“*In Diversity There is Epistemic Strength*”—Helen Longino

It is not news that Science, Technology, Engineering and Math (STEM) disciplines are severely lacking wide representation from people belonging to non-White, ethnic and cultural groups of scientists (Tilghman et al., 2021). Efforts to increase diversity and inclusion in STEM fields are motivated by various concerns - a concern for equity and social justice (Byars-Winston, 2014; Coleman, 2020), and a concern for increasing the pool of scientists that are prepared to address contemporary needs in biomedicine, science and technology (Coleman et al., 2010; National Academy of Sciences 2011; Benish, 2018). Importantly, there is growing evidence that when scientists from diverse backgrounds and with unique experiences work together, a wider range of approaches to problem-solving are proposed and often more creative and groundbreaking solutions emerge (Hong and Page, 2004; Valentine and Collins, 2015). Our efforts to increase diversity in developmental biology and related fields are motivated by these factors. However, a fundamental concern intrinsic to scientific inquiry, is the reduction in objectivity in the scientific process and interpretation of data when our scientific community is less diverse. A key safeguard against this, is to have investigators from different backgrounds, experiences, ethnicity, sex, age, nationality, and so on, working on problems from different perspectives, using different methods and model systems. In other words, as Helen Longino (Longino Helen, 1990) and other philosophers and historians of science (Martin, 1991; Harding, 1992; Oreskes, 2019) have argued, if the diversity of the scientific community increases, the objectivity in generating new data and in its interpretation increases, leading to knowledge that is more reliable.

Science is an inherently a social endeavor that is affected by personal experiences (Longino Helen, 1990; Oreskes, 2019). These biases influence decisions on what, who and how to approach questions to find “truths” about our natural world. As in all disciplines, developmental biology has not been immune to narrow viewpoints supported by data generated from a largely homogenous community comprised of White males. Women and other marginalized groups have been largely left out, both in terms of being part of the scientific community and in the research process itself. Although, recently the number of White women scientists in developmental biology is currently similar to that of men, the consequences of this exclusion can be observed in the sexism underlying scientific accounts of reproductive biology and the roles of egg and sperm in fertilization (Longino Helen, 1990; Martin, 1991; Harding, 1992) or in the use of embryological and evolutionary findings to support sexism and racism such as those found in the eugenics movement (Longino Helen, 1990; Martin, 1991; Harding, 1992; Gilbert, 2001, 2021). Because values play an inevitable role, increased efforts to safeguard against current and future undetected biases leading to erroneous theories necessitates a scientific community with a rigorous peer review process that allows for criticisms and corrections influenced by differences in disciplinary experience as well as cultural and social viewpoints that comprise a diverse community of scientists within peer review groups.

Scientific societies are central to more than the peer review process. Professional societies organize and sponsor conferences that allow scientists to present their findings, vet new scientific ideas, and challenge those that are not sufficiently supported by evidence. These venues are invaluable platforms for all attendees to expand their disciplinary knowledge, meet, interact and form new professional networks, and create opportunities to pursue new scientific questions. These communities of scientists are one standard for how scientists work together not only to exchange and scrutinize ideas, but also to advocate and influence the science that will be subsequently published, presented nationally and internationally, and funded. Given the influence of scientific societies on the direction of scientific endeavors, the demographic of their membership is a serious concern to the success and collective impact of scientific research on society (COSSA, 2008; Madzima and MacIntosh 2021; Rushworth et al., 2021).

It is important to highlight differences between the demographics of the resident population in the UnitedStates in 2019 to the demographics in the STEM workforce (https://ncses.nsf.gov/pubs/nsf21321/data-tables). These data show that in 2019, the percent of Hispanics/Latinos in the UnitedStates was 18.45% but only 8.76% were employed (age 75 years or younger at full- and part-time status) in STEM fields. Representation of Black/African Americans and American Indian/Alaska Natives in STEM fields (6.89 and 0.32%, respectively) was also lower than their representation in the country (12.54 and 0.74%, respectively). In contrast, in 2019 Asians made up 13% of our STEM workforce, which is 2.25 times their representation in the UnitedStates population (5.76%). The representation of Whites, Native Hawaiian/Pacific Islander, and persons with disabilities working in STEM fields (68.45, 0.28 and 12.78%, respectively) was greater or somewhat closer to their representation in the country (60.11, 0.18 and 13.2%) than Hispanic/Latinos, Black/African Americans or American Indian/Alaska Natives.

In 2008, a consortium of various scientific organizations met to discuss the role of scientific societies in enhancing the diversity of those engaged in the sciences (COSSA, 2008). Their top recommendation was for scientific societies to make recruitment and retention of underrepresented (*UR*) scientists a goal, work with their membership and funding agencies to develop and sustain effective new initiatives, and monitor their impact aimed at broadening participation (COSSA, 2008). In 2012 the Society for Developmental Biology (SDB) was invited, along with a dozen or more other scientific societies, to a retreat sponsored by the National Science Foundation (NSF) BIO-Integrative Organismal Systems “Broadening Participation” initiative to discuss how NSF could work with scientific societies to increase the diversity in STEM. SDB was one of three scientific societies funded to test their pilot programs. In 2013, with funding from NSF, SDB established the *Choose Development!* Program.

The *Choose Development!* Program is focused on the recruitment and long-term retention of undergraduate students belonging to *UR* groups into a society of developmental biologists. The SDB *Choose Development!* Program provides fellows with a research-intensive experience equipped with a multi-tier mentoring plan, community-wide recognition and inclusion at national meetings, and continued tracking of their career progression. The early success of this program (Scientia, 2017) led to continued financial support from the SDB Board of Directors and donations from individual SDB members. Recently the National Institute of Child Health and Human Development (NICHD) has awarded a new grant to SDB to continue and expand this program given its success. Here, we report the outcomes of the *Choose Development!* Program as well as the positive effects on changing the infrastructure of the SDB and the diversity of its membership.

## Methods

Advertisement and recruitment of students and faculty for the *Choose Development!* (CD!) Program consist of contacting attendees at the annual meetings sponsored by the Society for the Advancement of Chicanos/Hispanics and Native Americans in Science (SACNAS) and Annual Biomedical Research Conference for Minority Students (ABRCMS) which target undergraduate; also by wide dissemination of flyers to the membership and through the SDB website, and at the SDB’s regional and national meetings every year. Active recruitment targets undergraduate students belonging to *UR* populations in STEM and attending a wide range of institutions (Junior Colleges, Primarily Undergraduate Institutions, etc.). Students and faculty complete separate applications that are due in early spring. These applications are made available through the SDB website (www.sdbonline.org/choose_development#Application). A three-person CD! leadership committee reviews all student and faculty applications and selects Fellows based largely on their academic performance, scientific curiosity, and interest in pursuing a research experience in developmental biology or related field. The students selected for the programs are referred to as CD! Fellows. Faculty are selected based on their record in mentoring students from diverse backgrounds, summer availability and research activity. Fellows are then matched to selected faculty based on mutual research interests and the Fellow is supported during two consecutive 10 weeks summer research internships.

Each Fellow is required to attend and present their summer research data during their second summer internship at the national SDB conference. Throughout their tenure in the program, Fellows are provided with multitude of professional development workshops and networking opportunities set up by the SDB that allow them to meet established developmental biologists via Zoom and in person when feasible. Various survey metrics have been used to assess the learning gains in scientific knowledge, understanding of the research enterprise, communication skills, attitudes towards pursuing graduate degrees and remaining in scientific research by the Fellows. The program has continued to seek updates from the Fellows after their CD! support. Mentors are surveyed to assess their mentoring experience, skills and attitudes in participating in the *Choose Development!* Program and its impact in their lab. This feedback is taken into consideration and modifications to the program are made each year to continue to improve the experiences for the Fellows.

## Results and Discussion

**FIGURE 1:**
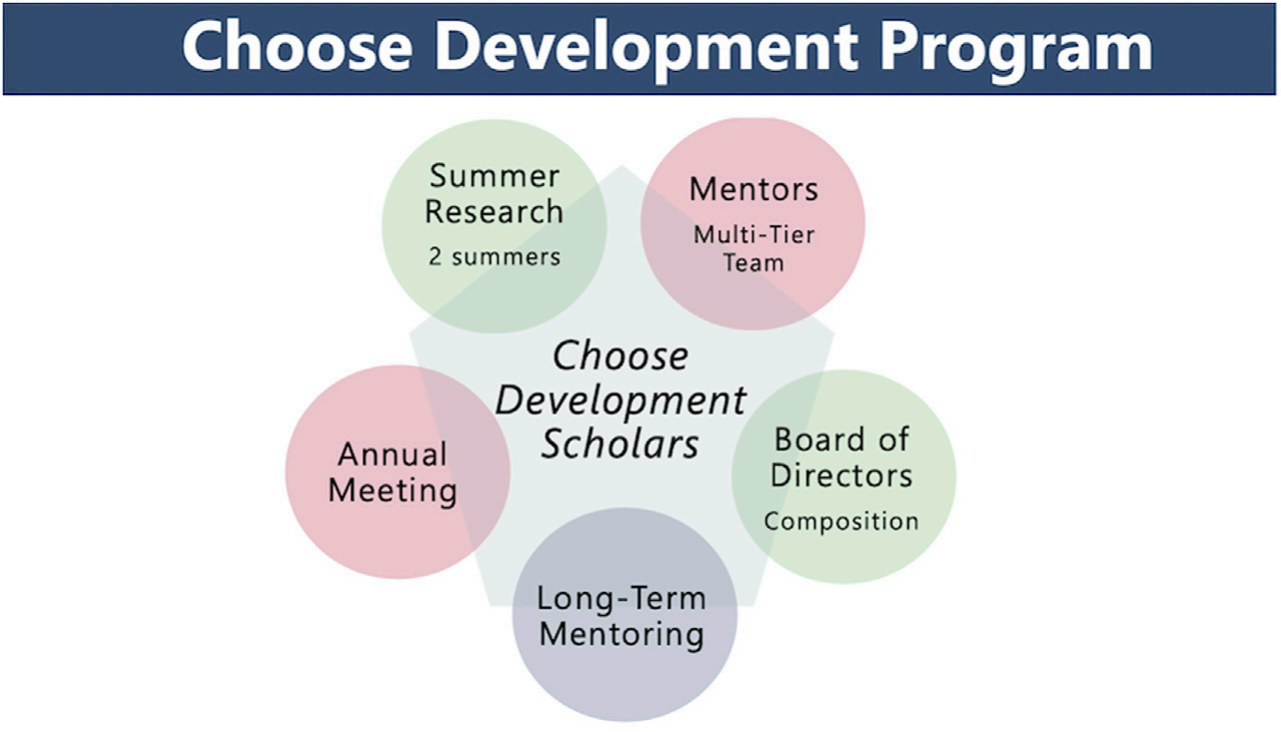
Transformation of the Society for Developmental Biology into a more diverse and inclusive scientific society through a comprehensive research-intensive experience and long-term mentoring of undergraduate students.

SDB was founded in 1939 as the Society for the Study of Development and Growth. In 1965, the name was changed to the Society for Developmental Biology. It took 35 years before the first woman, Dr. Elizabeth Hay, was elected president of the society in 1974 and 81 years to elect a president of Latino ethnicity, Dr. Alejandro Sánchez Alvarado (https://www.sdbonline.org/sdb_past_presidents). A voluntary survey of its membership in 2013 showed that individuals belonging to *UR* groups—Hispanic/LatinX, Native Hawaiian or Pacific Islander, Black or African American, American Indian or Alaska Native made up only about 10% of the SDB compared to 45.11% of the UnitedStates population (Table 1.2 in NCES Report, 2019). Members of the SDB Board recognized a need to increase the diversity of their membership, including *UR* groups and individuals with disabilities, across all academic levels. The drive to meet this goal led to the development of a comprehensive program called *Choose Development!*, which was established with funding by the National Science Foundation from 2013 to 2017 (https://www.sdbonline.org/choose_development). Here, we describe each of the society-wide initiatives (Figure 1) that has enhanced recruitment and engagement of established SDB members and has expanded inclusion of a wider range of scientific and social topics at our annual meetings that support diversity and inclusivity of all SDB members across social, cultural, ethnic and academic status, and institutional categories.

### Core Activity: A Two-Summer Research Intensive Experience and Presentation at the SDB Annual Meeting

#### Recruitment of Students Belonging to Underrepresented Groups to the Developmental Biology Community

Over the last 40 years many programs have been created to encourage *UR* individuals to pursue careers in science, engineering, and mathematics. Thus far, the assessment of short-term interventions to broaden participation in STEM has yielded low returns (see “Expanding Underrepresented Minority Participation: America’s Science and Technology Talent at the Crossroads” by NAS, 2011). A major challenge of these programs is helping *UR* students to see themselves as successful scientists. This is largely due to insufficient mentoring, lack of peer support, and a near absence of role models that represent the communities that these students are from (Sasso, 2008; Carnevale et al., 2011).

Recognizing that a strong research training experience in a laboratory setting that encouraged cooperative training and independent thinking had to be complemented with a strong and long-term mentoring support group (Handelsman et al., 2005), SDB developed the *Choose Development!* Program. This program provides a 2 year 10 weeks summer research immersion by *UR* undergraduate students in laboratories of established SDB members and encouraged by a strong multi-tier mentoring team throughout their tenure in the program and into their graduate programs. This multi-tier mentoring team is comprised of the Academic and Lab Mentors (see below) and the CD! leadership team. The training and mentoring structured around each Fellow were designed with the goal of increasing their professional network, sense of belonging in the community of developmental biologists, and likelihood of persisting in the sciences—an approach that has been validated by various groups since the inception of *Choose Development!* (NAS, 2011; Estrada, 2014; Estrada et al., 2016; Carpi et al., 2017; NAS, 2017; Martinez et al., 2018; Sellami et al., 2021).

*UR* students from 2 years Colleges, Primarily Undergraduate Institutions (PUIs) and Research (R1 and R2) institutions (Table 1) are encouraged to apply to this program. The SDB sponsored tables at national SACNAS and ABRCMS meetings where candidates were recruited for this program. In addition, CD! was presented to SDB members at their regional and annual meetings, in mailings to the entire membership, postings on websites of sister scientific societies, flyers sent to Minority Serving Institutions (MSIs), and direct communication with representatives who lead minority research training programs at their institutions.

**TABLE 1:** Demographics of *Choose Development!* fellows (2013–2020).

	American Indian	African American	Hispanic / Latino	Hawaiian / Pacific Islander	Female / Male	Students with disabilities
2-years College	-	-	2	-	1 / 1	-
PUI	-	2	1	-	2 / 1	-
R1 and R2	1	8	20	1	17 / 11	3

PUI: primarily undergraduate institution.

R1 and R2: research intensive university; Research University.

Some students self-identified as multi-racial or multi-ethnic.

As described above, undergraduate students who are beginning their sophomore or junior years with 2 years remaining in their baccalaureate degree are recruited through active outreach. The preferred applicants are those who belong to an *UR* group in STEM and are full-time students in good academic standing (minimum GPA of 3.0) who provide evidence of a strong interest and commitment towards pursuing a career in developmental biology or any related field as per their personal essay, research interests and letters of reference. Students from institutions where research experience is difficult to achieve are preferred, if academics are acceptable. Students with special needs are encouraged to apply, and a host lab that can provide the needed facilities sought. The cohort of Fellows chosen has ranged from ten to five, with the smaller numbers in years of limited bridge funding. This program has invested funds from the SDB, NIH and NSF on each Fellow’s team. The expenses include summer stipends for the Fellow, housing allowances when needed, and registration and help with travel funds to the annual meeting in the second year for the Fellow and a Mentor.

Once selected, the Fellows were matched with the lab of an established SDB member with shared research interests and a strong record of mentoring *UR* students in their research group. These SDB members could be at or away from the Fellow’s home institution. SDB members who wish to mentor a CD! Fellow also completed an application and underwent a thorough review. After each team is in place, Fellows and Mentors attend pre-summer workshops to ensure compliance with all programmatic requirements prior to starting their research projects. Upon joining their summer lab, Fellows are asked to make videos of their summer research experience in collaboration with their Academic Mentors. This activity bolsters the Fellow-Mentor relationship and communication from the beginning of their summer experience. These videos are uploaded to the SDB website and are used during the national meeting to introduce the Fellows to the entire society (https://www.sdbonline.org/choose_development_fellowvideos). During the summer, Fellows are required to complete and discuss an Individual Development Plan (IDP) with their Mentors. The IDP helps the Mentor become aware of the Fellow’s career goals, their experiences and assets, as well as the disciplinary training and professional skills needed by them to achieve their career goals.

### Outcome #1: Choose Development! Increased the Number of Underrepresented Undergraduates and Students With Disabilities Entering Graduate Programs in Developmental Biology

Between 2013 and 2020, *Choose Development!* accepted 33 undergraduate Fellows to its program. To date, 30 of 33 Fellows (91%) accepted into the Program have obtained their baccalaureate degree. As shown in Table 2, 19 (63%) of those that graduated have entered a graduate program in developmental biology or related biological fields (including one MD/PhD). In addition, 3 (10%) have entered medical school, two (7%) are employed in science-related fields, four (13%) have taken gap years to submit a more competitive application to graduate schools, and two (7%) have not responded to our annual surveys. Despite the relatively small number of Fellows to date, these are very high outcomes given both the graduation rates (91%) and persistence in science or science-related careers (80%, Table 2: all Fellows except those taking a gap year or are unresponsive).

**TABLE 2:** Academic/ professional status of *Choose Development!* fellows with Bachelor’s degree.

Total with BS /BA	Doctoral program	Master’s program	Medical school	Gap year app Grad	Industry	Unknown
30	15 (50%)	4 (13%)	3 (10%)	4 (13%)	2 (7%)	2 (7%)

#### Incorporation of Choose Development! Fellows into the SDB at Annual Meetings

**FIGURE 2:**
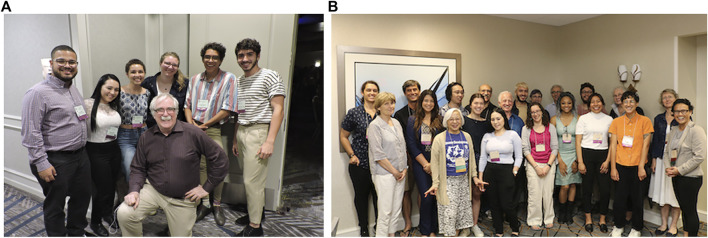
**(A)**: *Choose Development!* Fellows at the 2018 SDB Annual Meeting meet Nobel Laureate Dr. Eric Wieschaus (center). Fellows from left to right: Evan Brooks, Alexis Camacho-Avila, Amber Rock, Caroline Pritchard, Christian Wilson and Josean Reyes Rivera. **(B)**: *Choose Development!* Fellows at the 2019 SDB Annual Meeting lunch with renowned developmental biologists. From left to right: Lindsey Hernandez (2018); Ruth Lehmann; Richard Behringer; Adriana Vélez (2018–2019); Ida Chow; Qinan Hu (Lab Mentor); Talia Hart (2015); Martin Chalfie; Alexis Camacho-Avila (2017–2018); Mario Capecchi; Josean Reyes Rivera (2016, 2018); Rebecca Green (Lab Mentor); Brigid Hogan; Doug Melton; Grace Jean (2018); Davys Lopez (2013); Diana Ramirez (2018–2019); Christiane Nuesslein- Volhard; Amanda Neves (2018); Lilianna Solnica-Krezel; Graciela Unguez. Martin Chalfie, Mario Capecchi, and Christiane Nuesslein-Volhard are all Nobel Laureates; Ruth Lehmann, Brigid Hogan, Doug Melton and Lilianna Sonica-Krezel are all members of the National Academy of Science and SDB past presidents.

An important part of this program is the opportunity for the Fellows to showcase their summer research accomplishments by presenting a poster at the subsequent year’s SDB annual meeting. This activity provides the opportunity to welcome the Fellows into the community of developmental biologists. They also meet the Board of Directors at a reception exclusive for these Fellows. This is an important event as it provides the Fellows with access to the SDB leadership as well as an opportunity for the leadership to understand the impact of the program on the Society. Each year at the annual meeting the Fellows are also introduced to the community through different social events that include the opening reception, closing banquet, and a luncheon with distinguished, award-winning developmental biologists, including Nobel Laureates and members of the National Academy of Science (Figure 2). Their videos have often been shown during the meeting. The objective is to provide them with a sense that they have access to and support from the leaders in the developmental biology field as well as a sense that they can rely on the Society for long-term mentoring support. The long-range goal of the *Choose Development!* Program is for these students to flourish in the welcoming environment of the Society throughout their scientific careers. Such a system-wide commitment from a scientific society is both innovative and rare.

“*I think it has been very successful like again, walking around with (SDB faculty)*, *her introducing me to everybody has been very, very helpful. I mean I am getting really great resources and networks. And so it’s kind of fun because from going from the person I was in high school to where I am now is kind of … it’s still kind of surreal how supportive everybody has really been.*” --Former Fellow

### Complementary Activity 1: Long-Term Networking, Mentoring and Tracking

**FIGURE 3:**
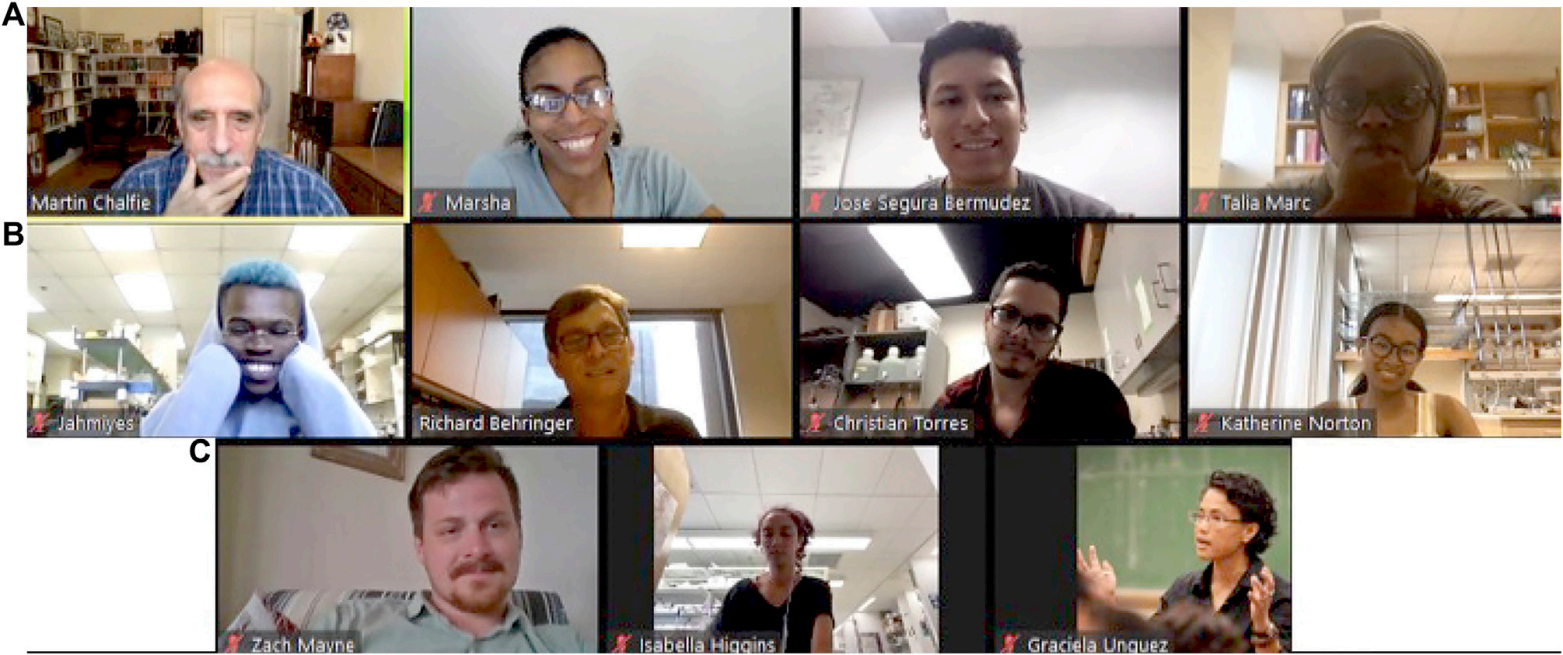
Screen picture of a weekly Zoom meeting with 2021 *Choose Development!* Fellows and guest, former SDB President and Noble Laureate Dr. Martin Chalfie. **(A)** Martin Chalfie, Marsha Lucas (SDB Publications and Communication Director), Jose Segura Bermudez, and Talia Marc. **(B)** Jahmiyes Wright, Richard Behringer (*Choose Development!* Program Coordinator), Christian Torres, and Katerine Norton. **(C)** Zach Mayne, Isabella Higgins, and Graciela A. Unguez (*Choose Development!* Program Director).

To ensure continued communication and tracking of our CD! Fellows, the SDB has explored several online platforms that allow individuals to share documents and set up networking and mentoring discussions. For example, between 2014 and 2016, a *listserve* for each group (i.e., Academic and Lab mentors) and Fellows (current and former), was generated in Trellis, a former online networking platform connecting different scientific communities provided by the American Association for the Advancement of Science. This allowed communication among current and former Mentors and Fellows so they could network and learn from each other as they transitioned out of the program and into graduate programs. Since 2017, the program has moved to the online Zoom platform to hold these meetings. This platform was essential to keep the Fellow-Mentor teams in contact during the summer of 2020 due to the COVID-19 pandemic. Online network forums are excellent sources for ongoing interactions between Fellows and Mentors and between Fellow-Mentor teams and the CD! leadership for evaluation purposes that lead to evidence-based modifications of the program. An innovative alternative to face-to-face introduction of the Fellows was started in 2020 by current program coordinator Dr. Richard Behringer, who recruited Nobel Laureates in the field, former and current SDB presidents and editors (current and former) of the official SDB journal *Developmental Biology* to meet current Fellows at weekly 1 h meetings during their 10 weeks summer research internship (Figure 3).

At these meetings, guests presented the Fellows with a wide range of professional trajectories and personal stories. The Fellows were provided many professional, research and networking opportunities. These weekly meetings proved effective in enhancing the Fellows’ circle of practitioners in the field, many of whom looked like the Fellows themselves and/or had cultural and geographical backgrounds similar to those of the Fellows. Moreover, these focused online scheduled meetings have provided Fellows from Primarily Undergraduate Institutions and Minority Serving Institutions with a considerably greater source of input and feedback from established developmental biologists, and a more expansive professional network available to these students than what they otherwise experience.

### Complementary Activity 2: *Choose Development!* Partners With the International Marine Biological Laboratory Embryology Course

Along with the two-summer research-intensive program, selected CD! Fellows were provided with the opportunity to visit the world-renowned Embryology Course at the Marine Biological Laboratory in Woods Hole, Massachusetts. Each summer, one or two Fellows were chosen to spend 1 week immersed in the course. This expanded their knowledge of developmental biology and new experimental techniques, while also providing an opportunity to network with national and international colleagues. To date, a total of ten Fellows have participated in this unique training opportunity. This exposure and integration of the Fellows into the wider national and international developmental biology community gave them a strong identity along with a supportive professional network of developmental biologists. Three of the Fellows who attended the Embryology course were invited to become course assistants for the Embryology Course in subsequent years. This additional experience cemented their sense of belonging with the developmental biology community beyond SDB, and all three Fellows are now pursuing their doctoral degrees.

### Complementary Activity 3: Implementation of Multi-Tier Mentoring Approach to Enhance the Training of Novice and Not-So-Novice Developmental Biologists

One of the most vital aspects to the success of this program is the multiple-tier mentor team format incorporated into the research and professional training of CD! Fellows. Each Fellow is assigned an Academic Mentor (Primary investigator/head of lab) and a Lab Mentor (postdoctoral fellow or an advanced graduate student) at the research lab. In turn, Academic and Lab mentors have a Master Mentor, an SDB member with extensive experience and record of training underrepresented students and postdocs in her/his lab, with whom to share concerns, request advice and discuss best practices in mentoring. In addition to exposing Mentors to inclusive communication skills and providing them with strategies to build an inclusive and culturally sensitive lab environment, the Lead Mentor facilitates discussions on implicit biases and approaches to optimize the sense of belonging in the lab of all Fellows.

*Choose Development!* provides all mentors, especially the senior faculty with a training workshop before the Fellows start the internship in their labs and at least one follow-up meeting to discuss programmatic issues and research progress of their Fellow. The feedback from these workshops has provided informed guidelines for best practices in mentorship which are disseminated to all SDB members and other scientific societies (e.g., Society for Neuroscience and AAAS) through meeting poster presentations. Because each Fellow must also be assigned a Lab Mentor, this program also offers professional development training to these early career scientists with the objective of providing them networking opportunities within the SDB community and skills essential for their careers as independent investigators. Incorporation of Lab Mentors has also elevated the awareness and reassurance of the Academic Mentors of their responsibility and role in cultivating these tiers of mentorship. Mentor workshops organized during the annual meetings give Academic and Lab mentors an additional opportunity to share and listen to other mentors’ experiences and obtain advice from more senior mentors. These active group discussions also encourage Mentors to share strategies to manage priorities and responsibilities in the lab.

The multi-tiered mentoring approach offered to Fellow-Lab Mentor-Academic Mentor teams is a unique approach in CD! and reinforces the culture of good mentorship practices that benefit the mentors and results in positive consequences for all the Fellows. A specific focus for Lab mentors is the honing of the following skills: 1) lab management and interpersonal skills, 2) communication skills, and 3) time management skills. In post-summer surveys, Lab Mentors commented on the structured multi-layer mentoring approach, which primarily manifested in positive impacts on their own growth as mentors, scientists, and communicators (Table 3). Academic Mentors reflected on how their communication style and skills impact the attitudes and prospects of undergraduate students continuing onto a graduate program (Table 3).

“*My participation was highly beneficial to my mentoring skills. My Fellow had a disability that I had little understanding of. This experience really taught me to explore different learning and communication styles with my mentees and to use campus resources, such as careers and disability advisors to manage expectations and career goals*.”—Former Lab Mentor

“*I am very grateful to be a participant in the Choose Development program. I think it is a wonderful initiative that is already having a tangible impact on student fellows and mentor participants. I look forward to more positive outcomes that will inevitably result from this program*.”

**TABLE 3:** Impact of *Choose Development!* program on academic and lab mentors—some insights.

Lab mentors	Academic mentors
The lab mentor continued to gain experience in mentoring students, in particular *organizing experiments to maximize the output of the mentee*. It also helped the lab mentor *think more deeply about his project*, by having discussions with the mentee.	I realized that undergrads greatly value *having discussions with me about the process of applying to graduate school…*
The Lab Mentor had to *learn how to manage his own time better*, so that he would be able to help manage the SDB Fellow.	Alerted me to my need to articulate aspects of science as a career that I may not be conveying well to all students - *the process, the rewards, the frustrations* - in addition to the nuts and bolts of the science…
The postdoc became *far more engaged in her own project*. She let the Fellow go with an aspect of the project that has fueled the next set of her experiments.	The Embryology course and the SDB meeting… showed [my SDB fellow] a wider world outside of my lab and our immediate environment… promoted his independence and inspired him to take more control over his project. *In the future I will promote these types of opportunities more*
Interacting with our Fellow really *brought his lab mentor out of her shell*. Having to support him and seeing his interactions with me *have given her more confidence in her own work and interactions with others in the lab*.	Figuring out how *to teach her to communicate effectively*
The Lab mentor *developed many new strategies for working with students with difficulties.* She was very inventive in some of the things she came-up with - and most of them did really help and I think will be useful to him in all aspects of his life.	It gave me the opportunity to *develop new strategies for mentoring students with difficulties*.

Former Academic Mentor

This mentor training plan has established the infrastructure within a national scientific society to support and foster the scientific enrichment of *UR* undergraduate students to successfully enter a graduate program and remain in the scientific research fields related to developmental biology. The introduction of this program to the SDB community raised awareness and appreciation for the benefits of good mentoring at all levels and of training a diverse population of future scientists. An atmosphere of inclusivity in the laboratories and classrooms is essential to foster and retain students who come from underserved and underrepresented populations. For their commitment and work efforts, all Mentors are recognized publicly at the annual SDB meeting for the commitment they have made to providing strong mentoring roles for the SDB Fellows. Several mentors have also participated in education workshops by sharing their experiences. Many established and not-so-established developmental biologists throughout the country who served as mentors or met the Fellows have since adopted the philosophy of long-term advocacy and mentorship.

### Outcome #2: Choose Development! Fellows Co-Author Presentations and Publications Based on Their Research Projects

A science identity is not complete until students fully participate in all aspects of professional scientific culture. This means they must understand the values of the profession they are joining and appreciate that their research is not complete until it is disseminated to the public through presentations at scientific meetings and/or publications. All CD! Fellows actualize the experience of being an active contributor to the production of scientific knowledge and its dissemination by submitting abstracts of their work and presenting it at poster sessions at the SDB Annual Meetings (2014–2020). Many of the 33 Fellows have also presented their research at the annual meetings of other scientific societies including SACNAS, ABRCMS, and the American Society for Cell Biology. Some Fellows have also been able to “professionalize” their summer research experience by engaging in the peer review and publication process of their results. To date, a total of 11 publications have involved the research conducted by Fellows, with nine publications having a Fellow as a co-author and two crediting Fellows in the acknowledgements for including research from their two research summers (Table 4). These accomplishments exemplify the level of dedication and commitment of the Academic Mentors toward the training and education of the Fellows, as well as the dedication and motivation of the Fellows.

**TABLE 4:** Research publications with data from *Choose Development!* fellows research projects.

2020
Martinez-Gómez, J., Galimba, K.D., Coté, E., Sullivan, A., Di Stilio, V.S. 2020. Spontaneous homeotic mutants and genetic control of floral organ identity in a ranunculid. *Evolution and Development* Special Issue (November 12, 2020). https://doi.org/10.1111/ede.12357
Hu, Q., Aviles-Velez, A., and Wolfner, M.F. 2020. Drosophila Plc21C is involved in calcium wave propagation during egg activation. *Micropublications Biology*
Hu, Q., Duncan, F.E., Nowakowski, A.B., Antipova, O.A., Woodruff, T.K., O’Halloran, T.T., Wolfner, M.F. 2020. Zinc dynamics during Drosophila egg maturation and activation. *iScience* 23(7): 101275.
Fellows acknowledged: Adriana Aviles-Velez and Lauryn Worley
2019
Wang, T.N., Clifford, M.R., Martinez-Gómez, J., Johnson, J.C., Riffell, J.C., Di Stiliio, V.S. 2019. Scent matters: differential contribution of scent to insect response to flowers with insect vs wind pollination traits. *Annals of Botany* 123(2), pp. 289–301
2018
Galimba, K.D., Martinez-Gómez, J., Di Stiliio V.S. 2018. Gene duplication and transference of function in paleo AP3 lineage of floral organ identity genes. *Frontiers in Plant Science*, 9, p. 334
Anna I Vickrey, Rebecca Bruders, Zev Kronenberg, Emma Mackey, Ryan J Bohlender, Emily Maclary, Raquei Maynez, Edward J Osborne, Kevin P Johnson, Chad D Huff, Mark Yandell, Michael D Shapiro. 2018. Introgression of regulatory alleles and a missense coding mutation drive plumage pattern diversity in the rock pigeon. *eLife* e34803. doi: 10.7554/eLife.34803
S. Basu, I. Barbur, A. Calderon, S. Banerjee, A. Proweller. 2018. Notch signaling regulates arterial vasoreactivity through opposing functions of Jagged1 and Dll4 in the vessel wall. *Am J Physiol Heart Circ Physiol*
Salinas-Saavedra, M., Rock, A.Q., and Martindale, M.Q. 2018. Germ layer specific regulation of cell adhesion: insight in to the evolution of mesoderm. *eLife* 7:e36740. doi: 10.7554/eLife.36740
Dubuc, T.Q. *, Stephenson, T.B.*, Rock, A.Q., and Martindale, M.Q. 2018. Hox and Wnt Pattern the First Primary Axis of an Anthozoan Cnidarian before Gastrulation. *Nature Communications* 9(1): (2018/05): 2007
2017
Pekar,O., Ow, M.C., Hui K.Y., Noyes, M.B., Hall, S.E., Hubbard, E.J.A. 2017. Linking the Environment, DAF-7/TGFβ signaling and LAG-2/DSL ligand expression in the germline stem cell niche. *Development* 144(16). pp. 2896–2906.
Fellow Acknowledged: Jesus Martinez-Gómez
2015
Sharma, P., Arazona, O.A., Lopez, D.H., Schwager, E.E., Cohn, M.J., Wheeler, W. and Extavour, C. 2015. A conserved genetic mechanism specifies deutocerebral appendage identity in insects and arachnids. *Proc Biol Sci*, Jun 7:282 (1808):20150698. doi:10.1098/rspb.2015.0698

NOTE: name of fellow in bold; name of Academic Mentor underlined.

### Complementary Activity 4: *Choose Development!* Spurs SDB Leadership to Better Represent Its Membership Needs

#### Restructure of the Board of Directors: Elected Office and Committees

Introduction of the CD! Program has led to expansion of inclusion and outreach of various groups within the Board of Directors (BOD). Specifically, the BOD approved the addition of three electable officers that would represent graduate students (2019), postdoctoral fellows (2020), and faculty at Primarily Undergraduate Institutions (PUIs, 2019) (https://www.sdbonline.org/board_of_directors). The first elected graduate student representative was a CD! Fellow, who recently received her PhD from Stanford University) and is now a postdoctoral fellow at UCSD in a developmental biology lab. Addition of these three new elected representatives to the BOD has greatly contributed to the BOD’s diversification, and most importantly, the contributions of the BOD has led to an expansion of inclusive activities that have influenced the structure of the SDB regional and national meetings

In 2012, two of fourteen BOD members and one out of nine members of two standing committees had *UR* status. Since inception of the CD! Program, SDB has elected its first Hispanic/Latino President (2019) and five BOD members that identify as African American or Hispanic/Latinos. In 2017, the Inclusion and Outreach Committee (IOC) was formed to oversee the design and implementation of program activities that promote “Development for all”. The goal of this campaign is to continue to message that SDB welcomes and supports anyone interested in developmental biology and related disciplines (https://www.sdbonline.org/ioc#mission). The IOC in conjunction with the SDB’s Professional Development and Education Committee (PDEC) has organized special symposia at the national annual meetings on unconscious bias, scientific bias, and mental wellness. The IOC also coordinates the offering of 1 h small group discussion led primarily by BOD members on topics requested by the SDB membership. These topics have included the following themes: networking for undergraduate and graduate students, applying to graduate schools and postdoc positions, preparing for academic and non-academic jobs, mentoring for trainees and faculty at all career stages, support for LGBQT+ members, and optimizing teaching and research at different types of institutions.

### Outcome #3: Changing Trends in Demographics of the SDB Membership

An increase in the number of SDB members that “look” like the Fellows will provide a more inclusive environment that facilitates the sense of belonging of the Fellows—an outcome that is essential for their progression and retention in the field of developmental biology. In 2012, the demographics from an SDB membership survey (voluntary participation) showed White members made up 78% of total respondents. Data from a more comprehensive database on the 2020 SDB membership shows a 9% decrease in the percentage (69%) of Whites compared to that in 2012 (Table 5). The total percent membership made up by underrepresented groups was similar in both 2012 (14.5%) and 2020 (14.43%). In these voluntary surveys, an increase was seen in the Hispanic/Latino membership (7.2 versus 9.30% in 2012 and 2020, respectively) and a decrease in members with disabilities (4 versus 0.016% in 2012 and 2020, respectively). The largest change was in the members that checked the “Other/Undisclosed” category—it more than doubled between 2012 (7.5%) and 2020 (16.06%).

**TABLE 5:** Demographics of the SDB membership (based on voluntary membership responses in 2012 and 2020)

	African American	American Indian	Hawaiian / Pacific Islander	Hispanic / Latino	White	Other / Undisclosed	People w/ Disabilities	Total
2012	16 (2.3%)	7 (1%)	0	49 (7.2%)	531 (78%)	51 (7.5%)	27 (4%)	681 (survey)
2020	52 (2.9%)	22 (1.2%)	5 (0.02%)	168 (9.30%)	1,242 (69%)	289 (16.06)	29 (0.016%)	1799 (database)

Broadening the representation of the SDB leadership has increased the active outreach to underrepresented scientists across the country to be speakers at regional and annual meetings. It has also led to an increase in *UR* candidates to the Nominating Committee for elected positions on the BOD. Collectively, the *Choose Development!* Program spurred the SDB to work towards maximizing the effectiveness of inclusion and diversity initiatives and promoting greater representation and participation in the Society by individuals belonging to *UR* groups. One of the most encouraging indicators of effective efforts by the entire SDB is the increase in the diversity of the undergraduate and graduate students joining the SDB since the inception of the CD! Program in 2013 (Table 6). Among these trainees, the percentage of Hispanics has doubled from 9.72% (2013) to 18.60% (2021). Although not as dramatic, the number of Black/African American (3.38 to 5.48%), American Indian (1.69 to 1.99%), and students with disabilities (1.69 to 2.16%) have also increased (Table 6).

**TABLE 6:** Demographics of trainees (undergraduate, graduate and postdocs) in the SDB

Year	Undergrad and Grad	Hispanic	Non- Hispanic	Undisclosed ethnicity	Black/African American	American Indian	Pacific Islander	White	Asian	Undisclosed race	Disability disclosed
2013	473	46 (9.72%)	369 (78.01%)	58 (12.26%)	16 (3.38%)	8 (1.69%)	4 (0.85%)	307 (64.90%)	91 (19.24%)	68 (14.38%)	8 (1.69%)
2014	571	51 (8.93%)	428 (74.96%)	92 (16.11%)	20 (3.50%)	9 (1.58%)	3 (0.53%)	311 (54.47%)	166 (29.07%)	85 (14.89%)	7 (1.23%)
2015	588	74 (12.79%)	444 (75.91%)	70 (11.90%)	23 (3.91%)	11 (1.87%)	1 (0.17%)	357 (60.61%)	134 (22.79%)	82 (13.95%)	4 (0.68%)
2016	660	69 (10.45%)	514 (77.88%)	77 (11.67%)	31 (4.70%)	8 (1.21%)	2 (0.30%)	412 (62.42%)	135 (20.45%)	94 (14.24%)	7 (1.06%)
2017	594	75 (12.72%)	470 (79.12%)	49 (8.25%)	28 (4.71%)	13 (2.19%)	6 (1.01%)	348 (58.59%)	155 (26.09%)	79 (13.30%)	10 (1.68%)
2018	583	75 (12.86%)	471 (80.79%)	37 (6.35%)	29 (4.98%)	9 (1.54%)	4 (0.69%)	360 (61.75%)	135 (23.16%)	69 (11.84%)	10 (1.71%)
2019	601	79 (13.14%)	471 (78.37%)	51 (8.49%)	35 (5.82%)	16 (2.66%)	5 (0.83%)	376 (62.56%)	137 (22.80%)	69 (11.48%)	10 (1.66%)
2020	703	104 (14.79%)	548 (77.95%)	51 (7.25%)	36 (5.12%)	18 (2.56%)	3 (0.43%)	412 (58.61%)	173 (24.61%)	99 (14.08%)	11 (1.56%)
2021*	602	112 (18.60%)	441 (73.26%)	49 (8.14%)	33 (5.48%)	12 (1.99%)	3 (0.50%	334 (55.48%)	158 (25.26%)	94 (15.61%)	13 (2.16%)

The cumulative outcomes of the CD! Program to date (Tables 2, 3) strongly support the positive impact of community-based long-term advocacy and mentoring of *UR* undergraduates in retaining students in a science career. Moreover, this Program has raised awareness and appreciation of the benefits reaped by all members across the entire SDB community when it champions structural changes that allow the inclusion and support of a diverse population of scientists into the Society. The high retention of CD! Fellows in scientific careers exemplifies an optimal convergence of highly dedicated and motivated *UR* students with a scientific community that is committed to support their successful research training and education. Although we highlight some promising outcomes, much work must still be done to ensure that these young scientists-in-training persist through the ranks of the academy. In order to ensure that the scientific process serves our nation and our society well, we must capitalize on the collective talents that people from all ethnic and cultural groups can bring to bear in solving the mysteries of the natural world.

Academic researchers are part of a society composed mostly of non-scientists who fund, participate in, benefit from, and in some cases are the subjects of scientific research. At the heart of the scientific endeavor is the conviction that when new knowledge is produced, these findings and their interpretations are reliable. This process warrants that research practitioners engage in a rigorous peer review process wherein scientists engage in presenting their findings, vetting ideas, and rejecting those that are not sufficiently supported by evidence prior to the publication of reports to the public. Because values play an inevitable role, diversifying our scientific community will more likely increase social practices of criticism, and corrections will detect unexamined assumptions, blind spots and inherited biases. But the need is even more fundamental because a diversity of ideas, which can originate from ethnic, cultural, and other forms of diversity, can enhance creativity and productivity, which is the very life blood of science.

A great case in point is that of Dr. Nettie Stevens whose research published in 1905 entitled “*Studies in Spermatogenesis*” affirmed that chromosomes play a role in determining sex during development, an idea that was against the more popular belief of that time that sex was determined by external factors (Brush, 1978). Due to her exclusion at scientific conferences because she was a woman, her findings were ignored and overshadowed by other more established male researchers, like Edmund Wilson who published a similar discovery using a different model of heredity and received all credit for Stevens’ original findings and conclusions.

Similarly, renowned embryologist Ernest Everett Just (1883–1941) emphasized the role that non-nuclear factors play in development and heredity. Specifically, he supported the view that embryonic differentiation was driven by all the parts of the cell, but especially the cytoplasm. This view was in sharp contrast to the emerging gene theory and nucleo-centric developmental processes put forth by Thomas Hunt Morgan and his followers (Manning, 1983; Sapp, 1998, Sapp, 2009). Although not all details of his view were correct, E.E. Just was correct about the importance of interactions between cytoplasmic factors and the nucleus (Manning, 1983; Sapp, 1998, Sapp, 2009). His work and views are worth revisiting within the context of his time and current knowledge that overwhelmingly questions genes and chromosomes as the sole basis of development.

E.E. Just’s insights and unique life experiences as an African American in the Academe were unlike those of Morgan’s whose patriarchal lineage included slave owners and Confederate General John Hunt Morgan (Sturtevant, 1959). E.E. Just’s drive to challenge Morgan was likely due to his ability to see things very differently than his peers (Manning, 1983; Byrnes and Eckberg, 2006; Byrnes and Just, 2015). Reflection on the important findings and interpretations of their work in developmental biology—both Nettie Stevens and E.E. Just were scientists whose careful observations and insightful interpretations were ignored and marginalized by the White male majority because of who they were—female or Black. As practicing research scientists, we recognize that exclusion practices of all scientific voices severely impair the scientific endeavor. We have helped spark a movement within the SDB to incorporate and establish inclusive practices in its education, training, networking and knowledge-sharing activities as detailed in this report. The momentum to recruit, attract and retain widely diverse scientific minds will lead to continued improvements in the quality of the scientific work we do. This is why diversity in science is important; critical scrutiny of all research done by scientists from different backgrounds leads to better collective knowledge. Understanding the evolution of our natural world is best done by the collective input of diverse developmental biologists

## Conclusions

To increase the diversity of experiences and backgrounds of individuals doing research in developmental biology and related fields, we have described ongoing efforts to improve the recruitment, retention, and inclusivity of individuals at all academic stages—from undergraduate to full professorship—in all activities sponsored by the SDB. Sparked by the *Choose Development!* Program first funded in 2013 by the NSF, the SDB has gradually expanded the multi-pronged approach by increasing the diversification of its Board of Directors membership, the speakers and institutions participating in the society’s regional and national meetings, widening the topics covered in professional development workshops to address broader needs, and galvanizing all members to fully engage in a scientific society that endorses its unofficial motto, “*Developmental Biology for All and by All*.”

It is not unrealistic nor impossible to make swift notable changes in how “we do science” within our niches to impact the number and diversity of individuals invited to be part of the scientific endeavor, peer review, decision making process and activities of a scientific society. The multi-pronged approach exemplified by the *Choose Development!* Program is one model by which a scientific community has encouraged and invited all its members to participate in helping make their community become more open, safe and inclusive environment for everyone. We have used *Choose Development!* to actively recruit underrepresented scientists for talks at meeting symposia, nominate these individuals for awards, and encourage them to run for elected office within the Board of Directors. Collectively working towards a more diverse and inclusive community of scientists where these young *Choose Development!* Fellows see themselves not just as a resource but rather the lifeblood that constitutes science itself is crucial for their success and for the scientific endeavor. All scientific communities can achieve effective inclusion and diversity. Creating an inclusive culture is not a one-off initiative. It is about a clear narrative that building diversity and maintaining it necessitates ongoing support, mentorship and governance. The pay-off of this *Choose Development!* program, is the creation of a more colorful and inclusive space that is pushing the frontiers of developmental biology in exciting directions.

## Data Availability

The original contributions presented in the study are included in the article/Supplementary Material, further inquiries can be directed to the corresponding author.
